# Isolation and molecular characterization of genotype 1 Japanese encephalitis virus, SX09S-01, from pigs in China

**DOI:** 10.1186/1743-422X-8-472

**Published:** 2011-10-14

**Authors:** Qi S Cao, Xiang M Li, Qiao Y Zhu, Dan D Wang, Huan C Chen, Ping Qian

**Affiliations:** 1 State Key Laboratory of Agricultural Microbiology, Huazhong Agricultural University, Wuhan city 430070, Hubei Province, P. R. China; 2 Laboratory of Animal Virology, College of Veterinary Medicine, Huazhong Agricultural University, Wuhan city 430070, Hubei Province, P. R. China

## Abstract

**Background:**

Pigs play a critical role in Japanese encephalitis virus (JEV) transmission between mosquitos and humans. In 2009, lots of piglets developed symptom of viral encephalitis in a pig farm in Yunchen, Shanxi province.

**Methods:**

Virus isolation was carried out in BHK-21 cells. Immunohistochemistry, RT-PCR and indirect immunofluorescent assay were used to identify the newly isolated virus. The complete genome of one isolate (SX09S-01 strain) was sequenced and analyzed. Two phylogenetic trees were constructed on the basis of the 24 full-length JEV genomes and 62 E genes mostly selected from China.

**Results:**

JEV SX09S-01 strain was isolated from piglets. Sequence analysis indicates that the completed genome sequences of this strain consists of 10965 nucleotides and there are 13 nucleotides deletion in the 3' nontranslated variable region. Compared with other JEV strains, homology ranges from 99.1% (XJ69) to 74.1% (XZ0934) and 99.6% (XJ69) to 91.1% (XZ0934) on the level of nucleotide and amino acid sequences, respectively. Phylogenetic trees show that SX09S-01 strain belongs to genotype I and it is most closely related to the XJ69 strain.

**Conclusions:**

Genotype I of JEV still circulates in Yuncheng and it is thus important for active surveillance on genotype I of JEV from the swine population.

## Background

Japanese encephalitis (JE) is mostly prevalent in eastern and southern Asia, such as China, India and Japan [1,2]. JE has extended its geographic regions and it has been recently reported in Australia and USA [2,3]. There are approximately 30,000-50,000 JE cases and up to 10,000-15,000 deaths occur worldwide each year [2,4]. In fact, the actual number of cases caused by Japanese encephalitis virus (JEV) is most likely to be much higher, and up to 175,000 each year [5]. Most of cases occurred in the young children and older people. Approximately 25%-30% of cases caused by JEV are fatal, and 50% result in permanent neuropsychiatric sequelae [2,6].

In China, JE is one of the most important viral encephalitis and has been reported in most provinces of mainland China, except for Xinjiang Uygur Autonomous Region and Qinhai Province [6,7]. There are still 8,000-10,000 cases reported annually and nearly 80% of globally reported cases occur in China although JE vaccines are widely used [8]. Particularly, in August 2006, an outbreak of JE occurred in Yuncheng, Shanxi Province, China Sixty-six human cases were reported with 19 fatalities [9].

JEV, the etiological agent of JE, belongs to a mosquito-borne *Flavivirus *within the family *Flaviviridae *[10]. JEV exists in a zoonotic transmission cycle between animals/water birds and human by *Culex *mosquitoes, and humans are a dead-end host [11]. The viral genome is a single-stranded positive sense RNA molecule approximately 11 kb in length and contains a single long open reading frame (ORF) that encodes a polyprotein flanked by 5' and 3' nontranslated regions (NTRs). The polyprotein consists of three structural proteins, designated capsid protein (C), membrane (M, a mature form of its precursor protein prM), and envelope protein (E), as well as seven nonstructural proteins (NS1, NS2A, NS2B, NS3, NS4A, NS4B and NS5). The prM and E proteins play critical roles in several biological activities, such as hemagglutination, neutralization, viral binding to cellular receptors and membrane fusion. In addition, the E protein has a major role in determining the neuorovirulence or neuroinvasiveness. Phylogenetic analyses mainly focused on partial sequences derived from either the C/prM or E gene and JEV can be divided into five genotypes (GI-GV) based on the nucleotide sequence of E gene [1,7,12].

Swine is an important reservoir and overwintering host for JEV and thus plays a critical role in the human encephalitis epidemics. However, the detailed information on JEV from pigs was lacking. Here, we report the isolation of a GI JEV from piglets that developed viral encephalitis in Yuncheng in July 2009 and this virus is designated SX09S-01 strain. To investigate its molecular characters, the completed genome was sequenced and analyzed. Eight critical amino acids in the E protein were found to be closely related to the JEV neurovirulence. Phyogenetic trees were constructed on the basis of the 24 full-length JEV genomes and 62 completed E genes mostly selected from China. Phyogenetic analysis indicated that SX09S-01 strain was most closely related to the XJ69 strain.

## Materials and methods

### Collection of samples and virus isolation

Seven swine brain samples were collected from piglets which were suspected to be infected with JEV in Yuncheng, Shanxi province 2009. A part of brains was fixed with 4% paraformaldehyde for immunohistochemistry and others were ground and used for virus isolation.

The virus was isolated on BHK-21 cells as previous described [13]. Briefly, the supernatant was inoculated onto the monolayer of BHK-21 cells, and the cytopathic effects (CPE) was observed daily under a microscope. Culture supernatants were harvested and re-inoculated onto fresh BHK-21 cells until the typical CPE of JEV appeared. One virus was isolated and designated SX09S-01 strain.

The SX09S-01 strain was further identified by indirect immunofluorescent assay (IFA). SX09S-01 was inoculated on BHK-21 cells monolayer, and IFA was carried out 36 hours later as previously described [14]. The primary antibody is murine monoclonal antibody against JEV E protein and the secondary antibody is FITC conjugated anti-mouse IgG (Cat no: BA1101, Boster).

### Virus purification

The SX09S-01 strain was propagated by injection into the brains of suckling mice [15]. The brain tissue was collected and ground while the first signs of paralysis emerged. The sample was centrifuged at 10000r/min for 30min and the supernatant was stored at -80°C as a virus stock. Mice were bought from Center for Animal Disease Control, Hubei province and all experiments were conducted with approval of the Animal Committee of Huazhong Agricultural University.

### Viral RNA extraction

Total viral RNA was extracted from the supernatant of infected cells with TRIZOL^® ^Reagent (Invitrogen, USA) according to the manufacturer's protocol. Briefly, 200 μl of sample was mixed with 1.0 ml of TRIZOL^® ^reagent and 0.2 ml of chloroform. After incubation on ice for 10 min, the aqua phase was separated by centrifugation (12,000 × g for 15 min) at room temperature (RT) and 500 μl of isopropyl alcohol was mixed with 80% of the aqua phase in a fresh tube. After incubation at RT for 10 min, the RNA was precipitated at 12,000 × g at RT for 10 min. After washing once with 80% ethanol, the pellet was briefly dried at RT and then dissolved with DEPC treated water and stored at -80°C until use.

### Full-length genome sequence analysis of the SX09S-01 strain

Extracted viral RNA was used as a template for cDNA synthesis using avian myeloblastosis virus (AMV) reverse transcriptase (Toyobo, Japan). The cDNA was subsequently used for PCR amplification with LA PCR™ Kit Ver.2.1 (TaKaRa, Dalian). In order to amplify the completed genome, the six pairs of primers (Table 1) were designed according to the XJ69 strain genome sequences. The PCR products were purified by agarose gels electrophoresis and then ligated directly into TA cloning vector system (Promega, USA) and sequenced. Analysis of nucleotide and deduced amino acid sequence identifies was performed by DNASTAR software.

**Table 1:** Primers used in this study for amplification of the full-length genome of SX09S-01 strain.

Segments	Primers	Oligonucleotide sequence (5'-3')	Position^a^	Length (bp)	Annealing temperature (°C)
S1	P1S^b^	5'--- AGAAGTTTATCTGTGTGGACTTC---3'	1-23	1795	58
	P1R^c^	5'--- CCACCACGATGGCTCCTGC---3'	1777-1795		
S2	P2S	5'--- TGYTGGTCGCTCCGGCTTA---3'	956-974	1582	57
	P2R	5'--- AAGATGCCACTTCCACAYCTC ---3'	2517-2537		
S3	P3S	5'--- GACACTGGATGTGCCATTG---3'	2479-2497	2310	57
	P3R	5'---TCTTTTTGTTGTTTTTAAAG---3'	4589-4608		
S4	P4S	5'--- GTTGGACGACGACGGCGACTTTC---3'	4449-4471	2648	58
	P4R	5'--- AGGTGATTAGGTGCTTCAGGAGAGG---3'	7072-7096		
S5	P5S	5'--- GGTCGGAGTGGTGGCAGCAAATG---3'	6894-6916	2200	58
	P5R	5'--- CCCTTTAGCCTTTCCGAACTCTCCG---3'	9069-9093		
S6	P6S	5'--- AGGAGTCAAGGAAGTGCTCAACG---3'	8787-8809	2178	58
	P6R	5'--- AGATCCTGTGTTCTTCCTCACCACC ---3'	10940-10964		

^a ^Numbers indicate the location of the nucleotide sequence of XJ69

^b ^S represents the sense primer.

^c ^R represents the anti-sense primer

### Multiple alignments and phylogenetic analysis

The JEV strains used in multiple sequence alignments and phylogenetic analyses in this study are listed in additional file 1. Multiple sequence alignments and phylogenetic analysis were performed by the neighbor-joining (NJ) method using the Maga4.1 software. To generate the rooted trees, the Murray Valley encephalitis virus (MVE-1-51) was used as an outgroup in phylogenetic analysis.

## Results

### Viral isolation

**Figure 1:**
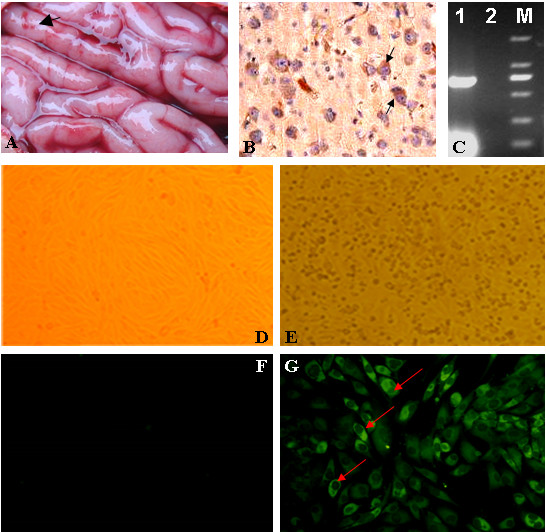
**Isolation and identification of the SX09S-01 strain**. (A) Lesions in the brain of piglet. Haemorrhage spots are indicated by filled arrow. (B) Immunohistochemistry (IHC) of the piglet's brain used the special monoclonal antibodies against JEV E protein. Brown cells, positive cells infected by JEV, are shown by the arrow. (C) RT-PCR results. Lane 1: 674-nucleotide fragment was amplified by special primers of the C/prM; lane 2: Negative control; lane M: DNA marker DL 2000, size of bands are 2000 bp, 1000 bp, 750 bp, 500 bp, 250 bp and 100 bp from top to bottom. (D) & (E) Cytopathic effect (CPE) of JEV in BHK-21 cells. Fig D is normal BHK-21 cells control. (F) & (G) Indirect immunofluorescent assay (IFA) with the specific monoclonal antibody against JEV E protein. Bright fluorescence cells were indicated by arrows. Figure G is normal cells control.

In July 2009, lots of piglets developed the viral encephalitis and died in Yuncheng, Shanxi province, China. Brain samples were collected and haemorrhage lesions were found (Figure 1A). To investigate whether it was caused by JEV, specific IHC and RT-PCR were performed. IHC revealed brown positive cells in the brain (Figure 1B) and RT-PCR with the C/prM primers resulted in the amplification of a 674 bp band (Figure 1C).

In order to isolate the pathogen, brain samples were ground and the supernatant was inoculated onto the fresh BHK-21 cells. BHK-21 cells appeared rounding, shrinkage and dislodgment from the cell surface under a microscope on days 3-4 after inoculation, and typical cytopathic effect (CPE) of JEV was observed (Figure 1E). Cells infected by the isolate reacted with the specific monoclonal antibody against JEV E protein by IFA. Several focuses of bright fluorescence were observed in the cytoplasm compared with normal control cells (Figure 1G).

### Full-length nucleotide and deduced amino acid sequence analysis

To further investigate the molecular characters of the SX09S-01 strain, the completed genome RNA was amplified with several pairs of primers listed in Table 1. It contains 10965 nucleotides in length and encoded a 10299-nucleotide single ORF (3432 amino acid residues) flanked by a 96-nucleotide 5'-NTR and a 570-nucleotide 3'-NTR. The complete genomic sequence of SX09S-01 strain was compared with other 24 JEV strains available in GenBank (Additional file 1, indicated by boldface type). The results of multiple alignments showed that homology ranged from 99.1 (XJ69) to 74.1% (XZ0934), and 99.6(XJ69) to 91.41 (XZ0934) on the levels of nucleotide and amino acid, respectively. The SX09S-01 strain shares the highest nucleotide sequence homology with other strains of the GI with 97.8% (KV1899)-99.1% (XJ69), and amino acid sequence homology between 98.3% (KV1899)-99.6% (XJ69). There is further distance with the XZ0934 strain of the GV, 75.8% and 91.4% homology on the level of nucleotide and amino acid, respectively (Additional file 2).

**Figure 2:**
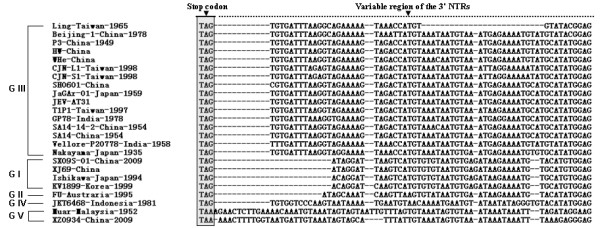
**Comparison of the nucleotide sequences in the variable region of the 3'-NTR of SX09S-01 strain with other 23 JEV strains**. The 66-necleotide sequences immediately downstream of the ORF stop codon of 24 JEV isolates, including sixteen GIII isolates, five GI isolates, one GII strain, one GIV strain and two GV, were aligned. Stop codon and variable region were showed on the top. Deletions are indicated by hyphens.

NTRs might play a vital role on the biological properties, including viral replication and neurovirulence. No nucleotide changes were found when compared with other three GI strains of JEV. However, there is an additional adenine (A) nucleotide insertion comparison with other genotype strains (data not shown). The nucleotide sequences of the 3'-NTRs of SX09S-01 strain was compared with those of other 24 strains belonging to GI to GV(Figure 2). A 13-nucleotide deletion immediately downstream of the translation stop codon was conserved among the 3'-NTR of the G I strains, including SX09S-01, the newly isolated strain. But a deletion of 11 nucleotides was observed in the FU strain of the GII. There was no nucleotide deletion found in 3'-NTRs of the JEV GIII strains, except for the Ling strain (25 nucleotides deletion). In addition, the 3'-NTRs of the JEV GVis about 14 nucleotides longer in than GII. The roles of these deletions in the 3'-NTR of the SX09S-01 strain needs further investigation.

### Analysis of the E gene amino acid sequences of SX09S-01 strains

JEV E protein is one of the major structural proteins and is closely related to viral virulence, host tropism and antigenicity. Eight amino acid residues of E protein play a critical role in the neurovirulence [16]. To analyze these key amino acids, we compared the E protein of SX09S-01 strain with SA14-14-2, an attenuated vaccine strain, and other virulent strains. No differences among the eight key amino acid residues were found between SX09S-01 and other virulent strains (Table 2). So, we predicted that the SX09S-01 strain possessed some typical characteristics of strong virulent JEV.

**Table 2:** Comparison of eight critical amino acids closely related to the neurovirulence of JEV in E protein between the virulent strains and the attenuated vaccine strain (SA14-14-2).

Strain	E107	E138	E176	E177	E264	E279	E315	E439
SX09S-01	Leu (L)	Glu (E)	Ile (I)	Thr (T)	Gln (Q)	Lys (K)	Ala (A)	Lys (K)
XJ69	Leu (L)	Glu (E)	Ile (I)	Thr (T)	Gln (Q)	Lys (K)	Ala (A)	Lys (K)
P3	Leu (L)	Glu (E)	Ile (I)	Thr (T)	Gln (Q)	Lys (K)	Ala (A)	Lys (K)
HW	Leu (L)	Glu (E)	Ile (I)	Thr (T)	Gln (Q)	Lys (K)	Ala (A)	Lys (K)
SA14	Leu (L)	Glu (E)	Ile (I)	Thr (T)	Gln (Q)	Lys (K)	Ala (A)	Lys (K)
SA14-14-2	Phe (F)	Lys (K)	Val (V)	Ala (A)	His (H)	Met (M)	Val (V)	Arg (R)

### Phylogenetic analyses

For better understanding of the genetic relationships and evolution of JEV strains, especially isolated in China, a total of the 62 sequenced JEV strains (shown in additional file 1) were used to construct phylogenetic trees. The genetic relationship was established on sequences of the full-length genome and the E gene sequences.

**Figure 3:**
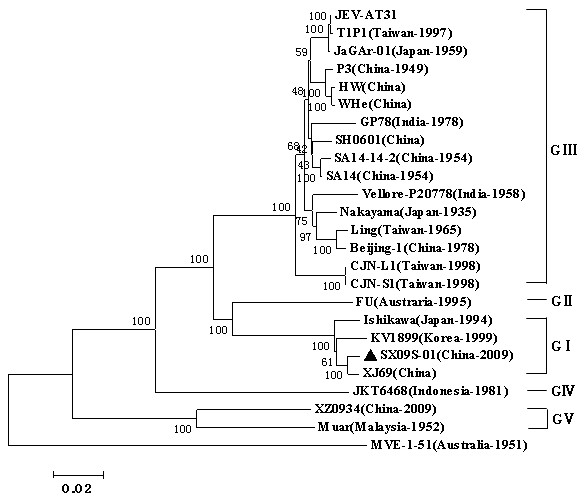
**Phyogenetic analysis of JEV strains based on the full-length genome**. The multiple sequence alignments were obtained by Mega4.1 software, and tree was constructed by the neighbor-joining method. Newly isolated strain, SX09S-01, was indicated by black triangle marker. Scale bar indicates number of nucleotide substitutions per site. Bootstrap confidence limits are shown at each node. The full-length genome sequence of the Murray Valley encephalitis virus (MEV) was used as an outgroup.

Phylogenetic tree was produced according to the full-length sequences by the NJ method. As shown in Figure 3, all the viruses could be classified into five groups and there were two distinct phylogenetic clusters. Newly isolated SX09S-01 is classified as JEV GI and this cluster also included the XJ69 (China), Ishikawa (Japan, 1994), and KV1899 (Korea, 1999) strains. SX09S-01 was closely related to the XJ69 strain isolated from *Culex tritaeniorhynchus *in China. The strains from India, China, Taiwan and Japan belonged to JEV GIII and be branched into four minor clades. The first clade consisted of early isolates from China (Beijing-1), Taiwan (Ling), India (Vellore-P20778) and Japan (Nakayama); The second contained the virulent strain (P3) and JaGAr-01 (Japan); The third included two strains from Taiwan (CJN-L1 and CJN-S1); other strains from India and China-including SA14 and its vaccine derivatives made up the fourth clade. Strain FU (Austraria, 1995) and JKT6468 (Indonesia, 1981) belonged the GII and GIV, respectively. GV consisted of two stains, Muar strain isolated from Malaysia in 1952 and XZ0934 strain recently isolated from China in 2009.

**Figure 4:**
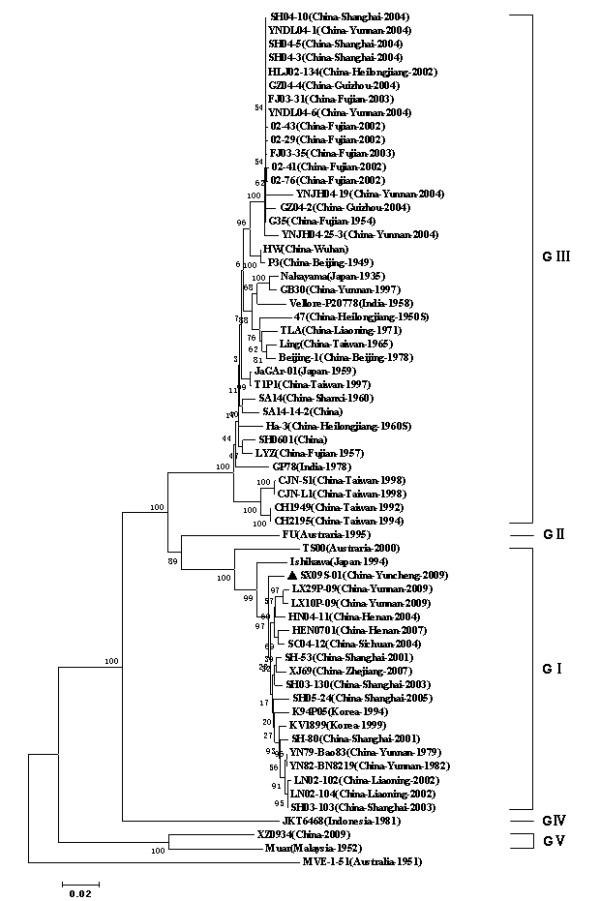
**Phylogentic tree was constructed by the neighbour-joining method based on the E genes of JEV strains mostly selected from different geographic regions in China at different time periods, where available in Genbank listed in additional file **1. Phylogenetic groupings corresponding to the genotyping classification are indicated from GI to GV. Newly isolated strain is indicated with black triangle marker. The scale at the bottom of the tree indicates the number of nucleotide substitutions per site. Murray Valley encephalitis virus (MEV) was used as an outgroup.

To determine the genetic relationships of JEV, the full-length E genes sequence was utilized to construct the phylogenetic tree. On the basis of the E gene sequences, a total of 62 JEV strains, which included 52 strains from China Mainland and Taiwan and 10 strains from other countries, were analyzed (Figure 4). Five distinct phylogenetic groupings were identified that corresponded to the five genotypes (GI to GV). The JEV strains isolated from China include GI, GIII and GV with the majority belonging to GI and GIII groups.

## Discussion

JE is mostly prevalent in China and there are many human JE cases reported recently [7,9,17]. However, there is very limited information on JEV strains originated from pigs. Pigs play a critical role to JEV transmission between mosquitos and humans, it is very important to surveillance the JE in the swine population in China.

In 2009, lots of piglets developed viral encephalitis in one pig farm in Yunchen, Shanxi province, where human JE cases broke out in 2006 [9]. In this study, we isolated a new JEV stain by serial passages on BHK-21 cells in Yunchen (Figure 1). The newly isolated JEV, designated SX09S-01, belongs to genotype I by C/prM sequences analysis. Tang [18] and Nerome [19] analyzed the molecular characterizations of JEV isolates from swine in Japan. To our knowledge, it's reported that only HEN0701 and SXBJ07 strains have been isolated from swine and belong to the GI JEV in China [20,21]. In this study, we clearly confirmed that GI JEV strains still circulate in Yuncheng. It suggests that we need to pay more attentions on the prevention and treatment of swine JE in order to control human JE effectively in this region.

To fully characterize the JEV SX09S-01 strain, its complete nucleotide and deduced amino acid sequences were determined. Compared with other strains isolated from different geographic regions at different time periods, a 13-nucleotide region that immediately followed the ORF stop codon was deleted in the genome of SX09S-01 (Figure 2). Similar deletions in this region were also observed in FU [1], K94P05 [22], Ishikawa strains (GenBank accession number AB051292), and Ling strain [23]. RNA secondary structure doesn't exist in this region by computational analyses [24,25]. Ta and Vrati [26] reported that a 60-nucleotide variable region immediately downstream of the ORF stop codon in JEV genome is not required for viral replication. However, it has recently been suggested that this variable region in the 3'-NTR may play an important role on the rate of viral RNA replication [22,27,28], but it still needs further clarification.

JEV E protein forms the viral spikes on the surface and has important biological functions related to virulence and viral host tropism. In this study, we compared the eight critical amino acids in E protein that are closely related with the neurovirulence of JEV with other virulent and attenuated vaccine SA14-14-2 strains and found that SX09S-01 strain has these typical characters of high virulent strain (Table 2) and displays high neurovirulence and low neuroinvasiveness in mice (Additional file 3). These results indicated that neuroinvasiveness of JEV may not have closely relations with the eight amino acids in the E protein and some other gene(s) contributed in part of the level of neuroinvasiveness. Nerome [19] reported that Sw/Mie/40/2004 is a high neurovirulent and neuroinvasive JEV and there were no amino acids differences in the E protein between Sw/Mie/40/2004 and other low neuroinasiveness strains. In addition, we also found that there was a censuses motif Arg-Gly-Asp (RGD) in the C-terminal of E protein. It has confirmed that protein contained RGD motif can interact with cellular integrins, such as VP1 protein of FMDV [29]. It is not known if JEV E protein interacts with intergrins for virus entry.

JEV can be divided into four genotypes based on a 240-nucleotide highly variable sequence of prM gene [9,30-32]. However, it results in unreliable information when short sequences (< 300 nt) were used in phylogenetic analyses of flaviviruses [33]. In recent years, full-length genome and E gene have also been used to establish phylogenetic trees for JEV [8,34-39] and JEV can be divided into five genotypes [7,12,40]. JEV E protein plays important roles in both induction of protective immune responses and in the biology of the virus [41,42]. In order to analyze the evolution of JEV in China, sequence information of JEV strains, originated from mainland China, were collected and phylogenetic trees were constructed on basis of the 24 full-length genomes or 62 E genes. Phylogenetic trees based on the full-length genomes or E genes provided similar topology (Figure 3, 4). JEV strains revealed five distinct phylogenetic groupings, reflecting broad geographical and temporal relationships. In China, JEV isolates are divided into three genotypes, GI, GIII and GV recently reported by Li MH et al [7]. GI strain was firstly isolated in 1979, whilst GIII strains have been isolated since the 1940s. Before the 1980s, GIII JEV was predominant in China. After 2000, there were more and more GIII JEV strains isolated and there is tendency that major genotype of JEV isolates changes from GIII to GI . In Japan, major genotype of JEV isolates had shifted from GIII to GI [19] and this phenomena were also found in Korea[39] and in Vietnam[43]. In mainland China, current vaccines used in human and pigs are inactivated or attenuated vaccines that both made up of the GIII JEV and it needs to investigate whether the same genotype shift will occur under the selected pressure of JEV vaccines.

In summary, here we have isolated GI JEV SX09S-01 strain from swine in Yuncheng in 2009. Its completed genome was sequenced and molecular characterization was analysized on the level of nucleotide and amino acid. Phyogenetic trees based on the full-length genome and E gene indicate that SX09S-01 strain is most closely related to the XJ69 strain. Future study should be aimed to investigate the efficacy of current vaccines against the SX09S-01 strain and other Chinese GI JEV strains and control the JE of pigs in order to prevent human JE.

## Conflict of interests

The authors declare that they have no competing interests.

## Authors' contributions

QSC, XML, QYZ carried out most of the experiments and drafted the manuscript. HCC and PQ critically revised the manuscript and the experiment design. DDW helped with the experiment. All of the authors read and approved the final version of the manuscript.

## Supplementary Material

Additional file 1 **Sources of the JEV strains used in the phylogenetic analysis in this study**. a Information unavailable. b Strain names labeled with bold world represent the full-length genome sequences available in GenBank. c Sequences used in the present study. Click here for file

Additional file 2 **Comparisons of the complete genomic sequence of the SX09S-01 strain with the sequences of 23 JEV strains available in GenBank**. 1-24: SX09S-01, Ishikawa, KV1899, XJ69, FU, CJN-L1, CJN-S1, GP78, HW, JaGAr 01, JEV-AT31, Ling, Nakayama, P3, SA14-14-2, SA14, SH0601, T1P1, Vellore P20778, WHe, Beijing-1, JKT6468, Muar, XZ0934. The percent nucleotide sequence identities of the complete genomes are presented at the upper right. The percent amino acid sequence identities of the complete genomes are shown in the lower left. The percentage of SX09S-01 sequence identities are indicated in boldface type. Click here for file

Additional file 3 **Comparative analysis of neurovirulence and neuroinvasiveness of the newly swine isolate and P3 strain**. a Examined by intracerebral inoculation with 30 μl. b Examined by intraperitoneal inoculation with 500 μl. Click here for file
