# Supplementary material for: Isolation and molecular characterization of genotype 1 Japanese encephalitis virus, SX09S-01, from pigs in China
Source: Virol J. 2011 Oct 14;8:472. doi: 10.1186/1743-422X-8-472 (PMC3213056; doi:10.1186/1743-422X-8-472)
Supplement: Additional file 1 — Sources of the JEV strains used in the phylogenetic analysis in this study. a Information unavailable. b Strain names labeled with bold world represent the full-length genome sequences available in GenBank. c Sequences used in the present study. [file 1743-422X-8-472-S1.DOC]

**Additional file 1: Sources of the JEV strains used in the phylogenetic analysis in this study**

| Strain name | place and year of isolation | soure | Genbank(E) |
| --- | --- | --- | --- |
| MVE-1-51 | Austraria,1951 | Human brain | NC-000943 |
| TS00 | Austraria,2000 | IU^a^ | EF434785 |
| K94P05 | Korea,1994 | IU | JEU34929 |
| **Ishikawa^b^** | **Japan,1994** | **IU** | **AB051292** |
| **KV1899** | **Korea,1999** | **IU** | **AY316157** |
| **FU** | **Austraria,1995** | **Human serum** | **AF217620** |
| **JEV-AT31** | **IU** | **IU** | **AB196923** |
| **JaGAr-01** | **Japan,1959** | **IU** | **AF069076** |
| **GP78** | **India,1978** | **Human brain** | **AF075723** |
| **Vellore-P20778** | **India,1958** | **Human brain** | **AF080251** |
| **T1P1** | **Taiwan,1997** | **mosquito** | **AF254453** |
| **CJN-S1** | **Taiwan,1998** | **IU** | **AY303793** |
| **CJN-L1** | **Taiwan,1998** | **IU** | **AY303794** |
| **Nakayama** | **Japan,1935** | **Human brain** | **EF571853** |
| **Ling** | **Taiwan,1965** | **IU** | **JEVLINGCG** |
| **JKT6468** | **Indonesia,1981** | **mosquito** | **AY184212** |
| 47 | Heilongjiang,1950s | Human brain | AY243827 |
| Ha-3 | Heilongjiang,1960s | Human brain | AY243842 |
| HLJ02-134 | Heilongjiang,2002 | Genus culicoides | DQ404081 |
| TLA | Liaoning,1971 | Human brain | AY243832 |
| LN02-102 | Liaoning,2002 | *Culex modestus* | DQ404085 |
| LN02-104 | Liaoning,2002 | *Culex pipiens pallens* | DQ404086 |
| **P3** | **Beijing,1949** | **Human brain** | **JEU47032** |
| HN04-11 | Henan,2004 | *Culex* | DQ404087 |
| HEN0701 | Henan,2007 | swine brain | FJ156730 |
| **SA-14-14-2** | **China** | **Vaccine** | **AF315119** |
| SC04-12 | Sichuan,2004 | *Culex* | DQ404090 |
| SH-53 | Shanghai,2001 | *Culex tritaeniorhynchus* | AY555757 |
| SH-80 | Shanghai,2001 | *Culex tritaeniorhynchus* | AY243841 |
| SH03-103 | Shanghai,2003 | *Culex tritaeniorhynchus* | DQ404096 |
| SH03-130 | Shanghai,2003 | *Culex tritaeniorhynchus* | DQ404104 |
| SH04-3 | Shanghai,2004 | *Culex tritaeniorhynchus* | DQ404105 |
| SH04-5 | Shanghai,2004 | *Culex tritaeniorhynchus* | DQ404106 |
| SH04-10 | Shanghai,2004 | *Culex tritaeniorhynchus* | DQ404107 |
| SH05-24 | Shanghai,2005 | *Culex tritaeniorhynchus* | DQ404108 |
| GZ04-2 | Guizhou,2004 | Armigeres | DQ404109 |
| GZ04-4 | Guizhou,2004 | Armigeres | DQ404110 |
| G35 | Fujian,1954 | Mosquito pool | AY243831 |
| LYZ | Fujian,1957 | Human brain | AY243834 |
| 02-29 | Fujian,2002 | Human cerebrospinal fluid | AY555762 |
| 02-41 | Fujian,2002 | Human blood | AY555763 |
| 02-43 | Fujian,2002 | Human blood | AY555764 |
| 02-76 | Fujian,2002 | Human blood | AY555765 |
| FJ03-31 | Fujian,2003 | Human blood | DQ404117 |
| FJ03-35 | Fujian,2003 | Human blood | DQ404118 |
| YN79-Bao83 | Yunnan,1979 | *Culex tritaeniorhynchus* | DQ404128 |
| YN82-BN8219 | Yunnan,1982 | *Culex tritaeniorhynchus* | DQ404129 |
| YNDL04-1 | Yunnan,2004 | *Culex tritaeniorhynchus* | DQ404137 |
| YNDL04-6 | Yunnan,2004 | *Culex pipiens quinquef ascitatus* | DQ404138 |
| YNJH04-19 | Yunnan,2004 | Unclassified Culex | DQ404147 |
| YNJH04-25-3 | Yunnan,2004 | *Culex tritaeniorhynchus* | DQ404148 |
| LX10P-09 | Yunnan,2009 | Homo sapiens | HM204528 |
| LX29P-09 | Yunnan,2009 | Homo sapiens | HM204529 |
| GB30 | Yunnan,1997 | Bat | FJ185037 |
| CH1949 | Taiwan,1992 | *Culex tritaeniorhynchus* | AF030549 |
| CH2195 | Taiwan,1994 | *Culex tritaeniorhynchus* | AF030550 |
| **HW** | **Wuhan** | **swine brain** | **AY849939** |
| **WHe** | **China** | **swine brain** | **EF107523** |
| **SH0601** | **China** | **IU** | **EF543861** |
| **XJ69** | **Zhejiang** | **IU** | **EU258742** |
| **Beijing-1** | **Beijing,1978** | **Human** | **L48961** |
| **SA-14** | **Shanxi,1960** | **Mosquito pool** | **M55506** |
| **SX09S-01^c^** | **China,2009** | **swine brain** | **HQ893545** |
| **Muar** | **Malaysia, 1952** | **Human** | **HM596272** |
| **XZ0934** | **China, 2009** | ***Culex tritaeniorhynchus*** | **JF915894** |

^a^ IU, Information unavailable.

^b^ Strain names labeled with bold world represent the full-length genome sequences available in GenBank.

^c^ Sequences used in the present study.
