# Supplementary material for: Isolation and molecular characterization of genotype 1 Japanese encephalitis virus, SX09S-01, from pigs in China
Source: Virol J. 2011 Oct 14;8:472. doi: 10.1186/1743-422X-8-472 (PMC3213056; doi:10.1186/1743-422X-8-472)
Supplement: Additional file 2 — Comparisons of the complete genomic sequence of the SX09S-01 strain with the sequences of 23 JEV strains available in GenBank. 1-24: SX09S-01, Ishikawa, KV1899, XJ69, FU, CJN-L1, CJN-S1, GP78, HW, JaGAr 01, JEV-AT31, Ling, Nakayama, P3, SA14-14-2, SA14, SH0601, T1P1, Vellore P20778, WHe, Beijing-1, JKT6468, Muar, XZ0934. The percent nucleotide sequence identities of the complete genomes are presented at the upper right. The percent amino acid sequence identities of the complete genomes are shown in the lower left. The percentage of SX09S-01 sequence identities are indicated in boldface type. [file 1743-422X-8-472-S2.DOC]

| **Isolate** | **% Sequence identity^a^** | | | | | | | | | | | | | | | | | | | | | | | | |
| --- | --- | --- | --- | --- | --- | --- | --- | --- | --- | --- | --- | --- | --- | --- | --- | --- | --- | --- | --- | --- | --- | --- | --- | --- | --- |
|  | **1** | **2** | **3** | **4** | **5** | **6** | **7** | **8** | **9** | **10** | **11** | **12** | **13** | **14** | **15** | **16** | **17** | **18** | **19** | **20** | **21** | **22** | **23** | **24** |  |
| **1** |  | **98.1** | **97.8** | **99.1** | **90.0** | **88.4** | **88.4** | **88.8** | **89.2** | **89.1** | **89.1** | **88.9** | **89.0** | **89.2** | **88.9** | **89.1** | **89.0** | **89.0** | **88.8** | **89.1** | **88.9** | **83.8** | **75.8** | **74.1** | **1** |
| **2** | **99.1** |  | 97.6 | 98.1 | 90.1 | 88.4 | 88.4 | 88.8 | 89.1 | 89.1 | 89.1 | 88.8 | 88.9 | 89.1 | 88.9 | 89.1 | 89.0 | 89.0 | 88.8 | 89.1 | 88.9 | 83.5 | 75.9 | 74.1 | **2** |
| **3** | **98.3** | 97.9 |  | 97.9 | 89.7 | 88.2 | 88.2 | 88.5 | 88.8 | 88.9 | 88.9 | 88.6 | 88.6 | 88.9 | 88.6 | 88.9 | 88.7 | 88.8 | 88.6 | 88.9 | 88.6 | 83.3 | 78.0 | 74.7 | **3** |
| **4** | **99.6** | 99.0 | 98.2 |  | 90.1 | 88.5 | 88.5 | 88.8 | 89.2 | 89.2 | 89.2 | 88.9 | 89.0 | 89.2 | 88.9 | 89.2 | 89.0 | 89.1 | 88.9 | 89.2 | 88.9 | 83.7 | 75.9 | 76.1 | **4** |
| **5** | **98.0** | 97.6 | 96.9 | 97.9 |  | 88.8 | 88.8 | 88.7 | 89.4 | 89.5 | 89.5 | 89.0 | 89.4 | 89.4 | 89.2 | 89.5 | 89.3 | 89.4 | 89.0 | 89.4 | 89.3 | 83.8 | 77.8 | 76.7 | **5** |
| **6** | **98.0** | 97.7 | 97.1 | 98.0 | 97.8 |  | 100.0 | 95.7 | 96.2 | 96.4 | 96.4 | 95.6 | 95.7 | 96.3 | 96.6 | 97.0 | 97.3 | 96.3 | 95.1 | 96.2 | 95.6 | 84.6 | 78.6 | 78.1 | **6** |
| **7** | **98.0** | 97.6 | 97.1 | 97.9 | 97.7 | 99.9 |  | 95.7 | 96.1 | 96.4 | 96.4 | 95.6 | 95.7 | 96.3 | 96.6 | 97.0 | 97.3 | 96.3 | 95.0 | 96.2 | 95.6 | 84.6 | 78.6 | 78.1 | **7** |
| **8** | **97.5** | 97.0 | 96.6 | 97.4 | 97.1 | 98.5 | 98.5 |  | 97.0 | 97.1 | 97.1 | 96.2 | 96.5 | 97.1 | 97.4 | 97.8 | 97.3 | 97.0 | 95.7 | 97.0 | 96.1 | 84.3 | 78.6 | 77.9 | **8** |
| **9** | **97.9** | 97.4 | 97.0 | 97.8 | 97.6 | 99.0 | 99.0 | 98.5 |  | 98.3 | 98.2 | 97.3 | 97.8 | 99.3 | 98.1 | 98.4 | 97.9 | 98.1 | 96.9 | 99.7 | 97.4 | 84.8 | 78.9 | 77.2 | **9** |
| **10** | **98.3** | 97.8 | 97.4 | 98.3 | 98.0 | 99.4 | 99.4 | 98.8 | 99.4 |  | 99.8 | 97.4 | 97.7 | 98.3 | 98.4 | 98.7 | 98.2 | 99.7 | 96.9 | 98.3 | 97.4 | 85.0 | 78.3 | 78.0 | **10** |
| **11** | **98.3** | 97.8 | 97.3 | 98.2 | 98.0 | 99.4 | 99.4 | 98.8 | 99.4 | 99.9 |  | 97.4 | 97.7 | 98.2 | 98.4 | 98.7 | 98.2 | 99.8 | 96.9 | 98.3 | 97.4 | 85.0 | 79.0 | 78.0 | **11** |
| **12** | **97.7** | 97.3 | 96.8 | 97.6 | 97.4 | 98.8 | 98.7 | 98.2 | 98.7 | 99.1 | 99.0 |  | 97.8 | 97.3 | 97.2 | 97.5 | 97.2 | 97.3 | 96.7 | 97.4 | 99.1 | 84.7 | 78.7 | 77.5 | **12** |
| **13** | **98.0** | 97.5 | 97.0 | 97.8 | 97.7 | 99.0 | 99.0 | 98.5 | 99.1 | 99.4 | 99.4 | 98.9 |  | 97.7 | 97.5 | 97.9 | 97.4 | 97.6 | 97.2 | 97.8 | 97.9 | 84.8 | 79.0 | 77.3 | **13** |
| **14** | **97.9** | 97.5 | 97.0 | 97.8 | 97.6 | 99.0 | 99.0 | 98.5 | 99.4 | 99.4 | 99.4 | 98.7 | 99.1 |  | 98.3 | 98.7 | 98.1 | 98.1 | 96.8 | 99.3 | 97.4 | 84.8 | 78.8 | 76.9 | **14** |
| **15** | **97.7** | 97.2 | 96.7 | 97.6 | 97.3 | 98.7 | 98.7 | 98.2 | 98.7 | 99.2 | 99.2 | 98.4 | 98.7 | 98.8 |  | 99.5 | 98.5 | 98.2 | 96.7 | 98.1 | 97.2 | 84.6 | 78.8 | 77.5 | **15** |
| **16** | **98.0** | 97.6 | 97.1 | 97.9 | 97.8 | 99.2 | 99.2 | 98.5 | 99.1 | 99.5 | 99.5 | 98.8 | 99.1 | 99.2 | 99.4 |  | 98.9 | 98.6 | 97.0 | 98.5 | 97.6 | 84.8 | 78.9 | 77.8 | **16** |
| **17** | **97.9** | 97.5 | 97.0 | 97.8 | 97.7 | 99.2 | 99.2 | 98.5 | 99.1 | 99.4 | 99.3 | 98.8 | 99.1 | 99.2 | 98.7 | 99.1 |  | 98.1 | 96.6 | 97.9 | 97.2 | 84.7 | 78.6 | 77.5 | **17** |
| **18** | **98.1** | 97.7 | 97.3 | 98.0 | 97.8 | 99.2 | 99.2 | 98.6 | 99.2 | 99.7 | 99.7 | 98.9 | 99.2 | 99.3 | 99.0 | 99.3 | 99.2 |  | 96.8 | 98.1 | 97.3 | 84.9 | 78.9 | 77.9 | **18** |
| **19** | **97.8** | 97.4 | 96.9 | 97.8 | 97.5 | 98.7 | 98.7 | 98.2 | 98.6 | 99.1 | 99.0 | 98.5 | 98.8 | 98.6 | 98.4 | 98.8 | 98.7 | 98.9 |  | 96.9 | 96.7 | 84.6 | 79.0 | 77.9 | **19** |
| **20** | **98.0** | 97.6 | 97.1 | 97.9 | 97.7 | 99.2 | 99.1 | 98.6 | 99.7 | 99.6 | 99.5 | 98.8 | 99.2 | 99.5 | 98.9 | 99.2 | 99.3 | 99.4 | 98.7 |  | 97.4 | 84.9 | 78.9 | 77.2 | **20** |
| **21** | **97.9** | 97.5 | 97.0 | 97.8 | 97.6 | 98.9 | 98.9 | 98.3 | 98.9 | 99.2 | 99.2 | 99.4 | 99.0 | 98.9 | 98.5 | 99.0 | 98.9 | 99.0 | 98.6 | 99.0 |  | 84.7 | 78.8 | 77.6 | **21** |
| **22** | **94.9** | 94.4 | 93.9 | 94.8 | 94.6 | 95.5 | 95.4 | 94.7 | 95.0 | 95.4 | 95.4 | 94.9 | 95.1 | 95.0 | 94.8 | 95.1 | 94.9 | 95.3 | 95.0 | 95.1 | 95.0 |  | 77.6 | 77.6 | **22** |
| **23** | **91.4** | 91.1 | 90.3 | 91.3 | 91.4 | 91.8 | 91.8 | 91.1 | 91.4 | 91.8 | 91.7 | 91.3 | 91.5 | 91.4 | 91.3 | 91.6 | 91.5 | 91.6 | 91.5 | 91.6 | 91.6 | 90.8 |  | 90.5 | **23** |
| **24** | **91.1** | 90.7 | 90.0 | 91.0 | 91.2 | 91.5 | 91.5 | 90.8 | 91.1 | 91.5 | 91.4 | 91.0 | 91.1 | 91.1 | 90.9 | 91.3 | 91.2 | 91.2 | 91.1 | 91.2 | 91.2 | 90.6 | 98.3 |  | **24** |
|  | **1** | **2** | **3** | **4** | **5** | **6** | **7** | **8** | **9** | **10** | **11** | **12** | **13** | **14** | **15** | **16** | **17** | **18** | **19** | **20** | **21** | **22** | **23** | **24** |  |

Additional file 2: Comparisons of the complete genomic sequence of the SX09S-01 strain with the sequences of 23 JEV strains available in GenBank

1-24: SX09S-01, Ishikawa, KV1899, XJ69, FU, CJN-L1, CJN-S1, GP78, HW, JaGAr 01, JEV-AT31, Ling, Nakayama, P3, SA14-14-2, SA14, SH0601, T1P1, Vellore P20778, WHe, Beijing-1, JKT6468, Muar, XZ0934.

^a^ The percent nucleotide sequence identities of the complete genomes are presented at the upper right. The percent amino acid sequence identities of the complete genomes are shown in the lower left. The percentage of SX09S-01 sequence identities are indicated in boldface type.
