# Supplementary material for: Isolation and molecular characterization of genotype 1 Japanese encephalitis virus, SX09S-01, from pigs in China
Source: Virol J. 2011 Oct 14;8:472. doi: 10.1186/1743-422X-8-472 (PMC3213056; doi:10.1186/1743-422X-8-472)
Supplement: Additional file 3 — Comparative analysis of neurovirulence and neuroinvasiveness of the newly swine isolate and P3 strain. a Examined by intracerebral inoculation with 30 μl. b Examined by intraperitoneal inoculation with 500 μl. [file 1743-422X-8-472-S3.DOC]

Additional file 3 Comparative analysis of neurovirulence and neuroinvasiveness of the newly swine isolate and P3 strain

| Virus strain | Neurovirulence^a^  (log_10_ p.f.u per LD_50_) | Neuroinvasiveness^b^  (log_10_ p.f.u per LD_50_) |
| --- | --- | --- |
| P3 | 0.16 | 4.16 |
| SX09S-01 | 0.62 | 5.87 |

^a^ Examined by intracerebral inoculation with 30µl

^b^ Examined by intraperitoneal inoculation with 500µl
